# Scaling human sociopolitical complexity

**DOI:** 10.1371/journal.pone.0234615

**Published:** 2020-07-02

**Authors:** Marcus J. Hamilton, Robert S. Walker, Briggs Buchanan, David S. Sandeford

**Affiliations:** 1 Department of Anthropology, University of Texas at San Antonio, San Antonio, TX, United States of America; 2 Department of Anthropology, University of Missouri, Columbia, MO, United States of America; 3 Department of Anthropology, University of Tulsa, Tulsa, OK, United States of America; 4 School of Human Evolution and Social Change, Arizona State University, Tempe, AZ, United States of America; Universidade Estadual de Maringa, BRAZIL

## Abstract

Human societies exhibit a diversity of social organizations that vary widely in size, structure, and complexity. Today, human sociopolitical complexity ranges from stateless small-scale societies of a few hundred individuals to complex states of millions, most of this diversity evolving only over the last few hundred years. Understanding how sociopolitical complexity evolved over time and space has always been a central focus of the social sciences. Yet despite this long-term interest, a quantitative understanding of how sociopolitical complexity varies across cultures is not well developed. Here we use scaling analysis to examine the statistical structure of a global sample of over a thousand human societies across multiple levels of sociopolitical complexity. First, we show that levels of sociopolitical complexity are self-similar as adjacent levels of jurisdictional hierarchy see a four-fold increase in population size, a two-fold increase in geographic range, and therefore a doubling of population density. Second, we show how this self-similarity leads to the scaling of population size and geographic range. As societies increase in complexity population density is reconfigured in space and quantified by scaling parameters. However, there is considerable overlap in population metrics across all scales suggesting that while more complex societies tend to have larger and denser populations, larger and denser populations are not necessarily more complex.

## Introduction

Human societies display a wide diversity of sociopolitical complexity. In the 21^st^ century, the smallest scales of social organization are politically autonomous hunter-gatherer families who self-organize into flexible, egalitarian groups of a few dozen individuals integrated into larger regional networks which form complex metapopulations that can include many hundreds of people [1–3]. For example, the Hadza of Tanzania are a hunter-gatherer population of ~1,000 people divided into four geographic regions [4]. Individual families form residentially mobile bands of fluid membership, usually consisting of ~20 individuals that fission and fuse with other bands over the course of a year [4]. Many small-scale societies still pursue predominantly subsistence lifestyles—whether forager, horticulturalist, pastoralist, or farmer—with varying levels of interaction with market economies, though there are still several dozen isolated populations on the planet with little to no effective interaction with the outside world [5–9]. The largest human organizations are complex states, often comprised of millions of people structured in space by hierarchical networks of cities, towns, villages, and farms, with diverse economies and nested political institutions. The United States, for example, has a population of ~327 million people divided into 50 states with a dozen cities of more than a million, and a multi-tiered political hierarchy from local governments to the federal government [10].

This range of sociopolitical diversity evolved only recently in human evolutionary history. Beginning with the development of agricultural food production ~11,000 years ago in the ancient Near East and elsewhere in various regions of the planet shortly thereafter, many populations who were previously mobile egalitarian hunter-gatherers incorporated agricultural foods into their diets and became increasingly sedentary, economically diverse, and politically non-egalitarian. The first complex states in the ancient world arose ~6,000 years ago in agriculturally productive regions of the planet, including southern Mesopotamia, Egypt, the Indus Valley, China, and later in the Americas and sub-Saharan Africa [11–14]. The upper tail of sociopolitical complexity we see today was fueled by the industrial revolution, which spurred unprecedented population growth, the expansion of global markets, urbanization, and increased rates of technological and scientific innovation, resulting in even greater economic, political, and cultural asymmetries among larger and ever more complex societies.

An axiomatic feature of the archaeological, ethnographic, and historical record is that more complex societies were once less complex: the earliest states were once regional polities that emerged from networks of local villages, which were formed by farmers who were hunter-gatherers prior to the adoption of domesticated plants and animals [15]. Similarly, the 195-member states of the United Nations each emerged from a long series of economic, political, and historical processes that integrated once politically-autonomous societies that emerged themselves from previously politically-autonomous entities, and so on. A conspicuous feature in the evolution of this diversity is the quantitative and qualitative nature of sociopolitical complexity across these different scales. Clearly, the United States is not simply a vast conglomeration of 327 million hunter-gatherers, nor is a Hadza hunter-gatherer band a microcosm of the Tanzanian state.

**Fig 1:**
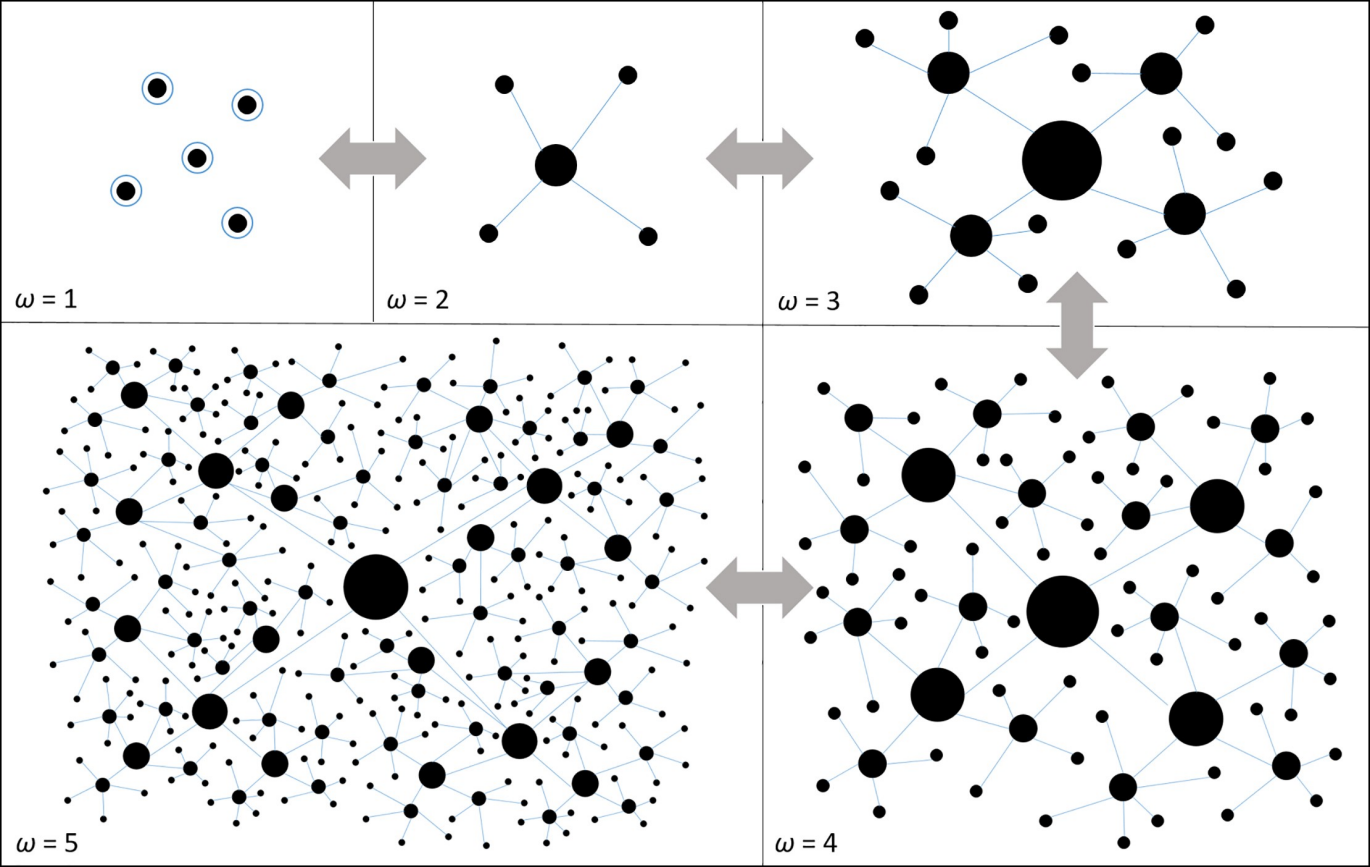
A schematic of the various scales of human sociopolitical complexity, from *ω* = 1, where individual communities are politically autonomous, and *ω* = 2 were local communities are bound together by an additional level of jurisdictional hierarchy denoted by the blue edges between nodes, up to *ω* = 5, the most complex state societies with five levels of sociopolitical and spatial hierarchy. In each panel there is an approximate 4-fold increase in the connected population size and an approximate 2-fold increase in population geographic range, and so a doubling of population density.

A central focus of anthropology over its history has been to understand the evolution of sociopolitical complexity [15]. An influential early model of sociopolitical complexity was the bands, tribes, chiefdoms, states hierarchy first proposed by Service in 1962 [16]. Ever since its first appearance in the anthropological literature this model was widely criticized as it reduces the enormous diversity of sociopolitical complexity into four discrete classes, arranged into an evolutionary hierarchy [17]. However, others have built on the model and view it as a useful conceptual framework as it captures basic qualitative and quantitative differences between societies that differ in sociopolitical organization [15]. While it is impossible to identify a set of robust criteria that successfully discriminates one category from all others, complex societies tend to have larger populations spread over broader geographic ranges with more political and economic institutions. The *Ethnographic Atlas* was published by Murdock in 1967 [18], and recently updated [19]. Originally, Murdock compiled data on 862 societies, now 1,264 [19,20]). The goal of this database was to allow researchers to conduct data-driven cross-cultural ethnological comparisons among a sample of human societies globally. An important metric of sociopolitical complexity in the *Ethnographic Atlas*–still used throughout the social sciences—is “the level of jurisdictional hierarchy beyond the local community” (variable 33) [21]. In effect, this level of sociopolitical complexity is similar to Service’s categorization, but uses a clearer definition: for each society the level of sociopolitical complexity, *ω*, ranges in scale from 1 to 5, where 1 is the minimal condition of a stateless acephalous society, such as many hunter-gatherer or subsistence-level agricultural societies, up to 5, a multi-tiered hierarchical complex state (Fig 1). Therefore, in level 1 there is no political authority recognized beyond the local community; at level 2 there are two-tiers of political authority, and so on up to level 5. As a metric, sociopolitical complexity continues play a fundamental role in comparative social scientific research. For example, the Seshat databank [22] is a recent attempt to estimate social complexity (and other metrics) in prehistoric societies using archaeological and historical data for statistical analysis [23–26].

A now well-documented feature of human social systems is the often modular and multi-tiered organization of social networks [2,13,15,27–29]. Examples include of hunter–gatherer social networks [2,27,30–32], small-scale autonomous village societies [29,33], the infrastructure of both ancient and modern cities [34–37], the internal organization of ancient states and empires [13], and the institutional infrastructure of modern nation-states [36,38–49]. Statistical signatures of these complex social structures are the constant branching structures indicative of self-similarity, and in the scaling behavior of social systems as they increase in size [50]. In this paper, we examine the organization of a global sample human societies across the spectrum of sociopolitical complexity. We examine population structure across the five levels of sociopolitical complexity using scaling statistics, including Horton-Strahler branching, generalized Horton Laws, and spatially-explicit mixed-effects models [2,51]. We focus on population size, *N*, the area of geographic range in km^2^, *A*, and population density in km^-2^, *D* = *N*/*A* both in terms of their average properties and their entire probability distributions.

### Branching ratios of population size and geographic range

**Fig 2:**
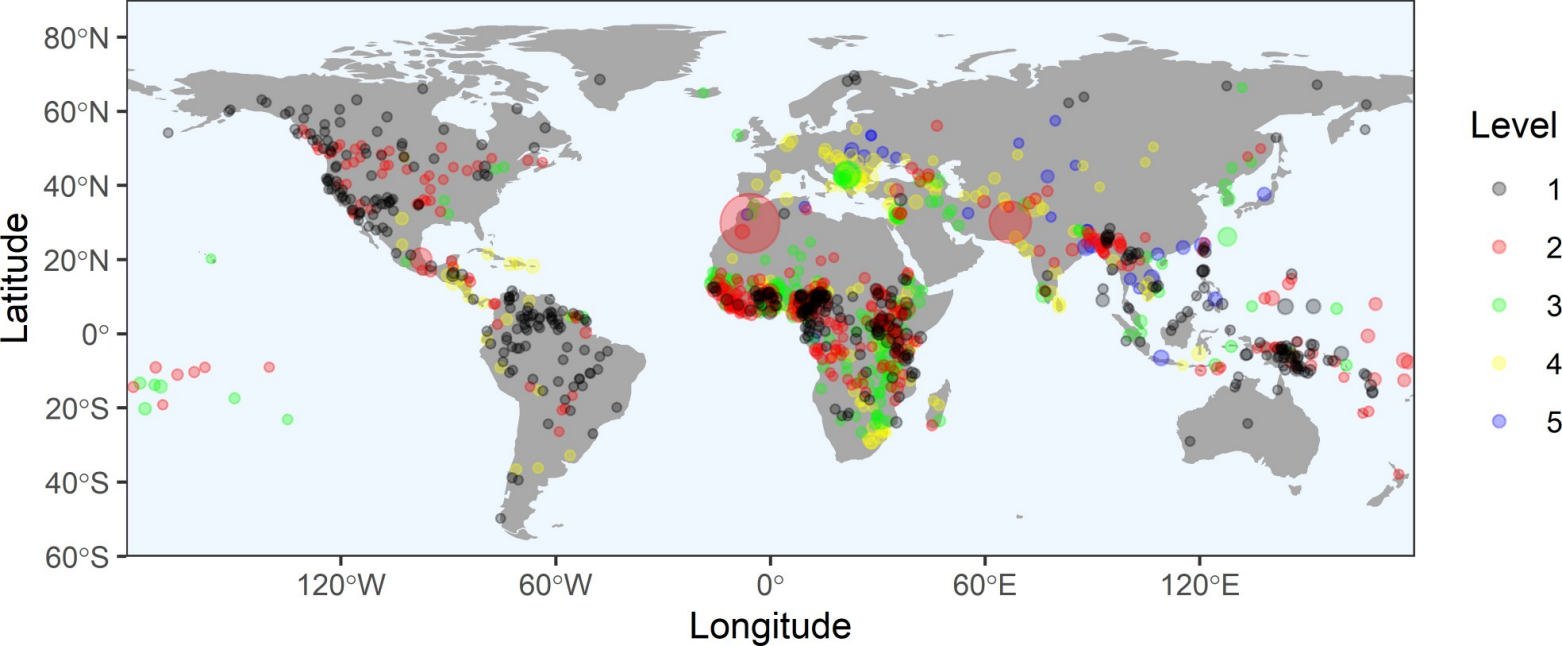
A world distribution map of the 1,121 traditional societies analyzed in this study color-coded by the level of sociopolitical complexity and scaled by population density. Black = 1; red = 2; green = 3; yellow = 4; blue = 5.

**Fig 3:**
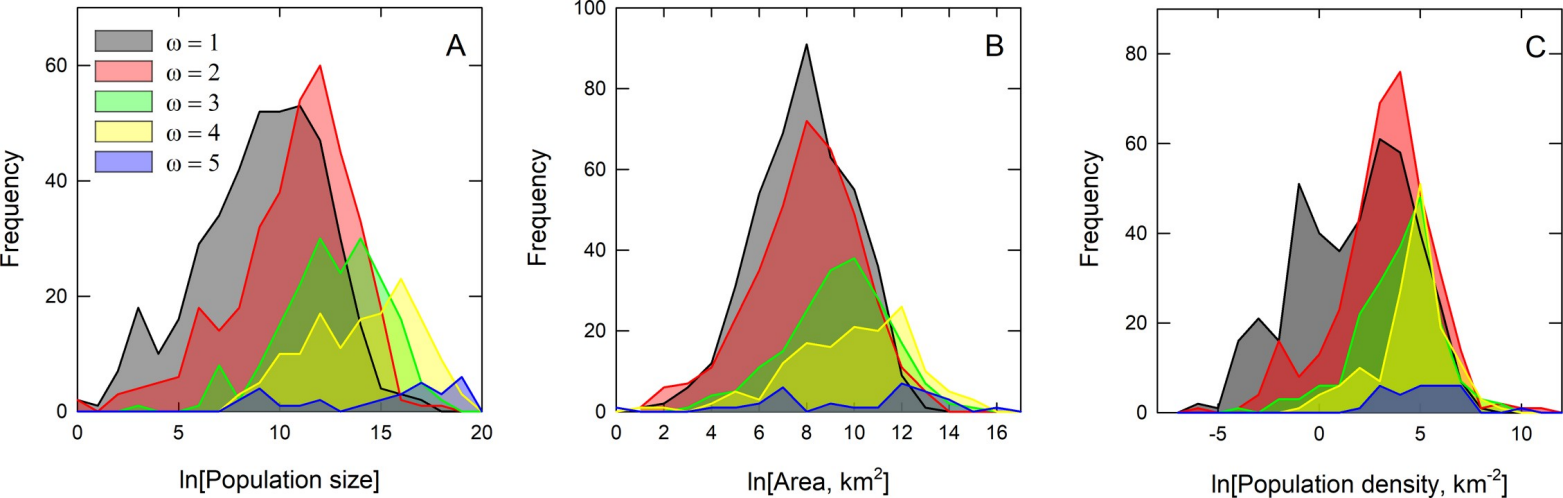
Frequency distributions of logged population sizes (A), geographic ranges (B), and population density (C) by sociopolitical level *ω*∈(1,5).

Fig 2 shows the global distribution of the 1,121 traditional societies used in the following analyses. First, we quantify sociopolitical levels by calculating the branching ratios of population sizes and geographic ranges from the data. Let *N*_*i*,*ω*_ be the size *N* of the *i*th population at level *ω*, and *A*_*i*,*ω*_ be the geographic range *A* (in km^2^) of the *i*th population at level *ω*. The mean sizes, variation, and confidence limits for the population size and geographic range data for each sociopolitical level are given in Table 1 and their distributions are shown in Fig 3, including population density. Average population sizes range from ~5,000 at *ω* = 1 to ~1.6 million at *ω* = 5, more than a 300-fold increase. Average geographic ranges range from ~1,600 km^2^ at *ω* = 1 to ~14,000 km^2^ at *ω* = 5, an 8-fold increase across the range. Given the distributions of population sizes, geographic ranges, and densities are approximately lognormally distributed (Fig 3, and see SI for statistical summaries) we use the multiplicative (or geometric) mean as the measure of central tendency. We first define ${\bar{N}}_{\omega }=\exp\left(\ln{\bar{N}}_{\omega }\right)$ as the mean population size at the *ω*th level, and ${\bar{A}}_{\omega }^{}=\exp\left(\ln{\bar{A}}_{\omega }\right)$ as the mean population geographic range at the *ω*th level. We then define the Horton-Strahler branching ratio, *R*_*N*_ as the ratio of means between levels:

$$R_{N}=\frac{{\bar{N}}_{\omega +1}^{}}{{\bar{N}}_{\omega }^{}}.$$ (1)

**Table 1:** Descriptive statistics for the population size and geographic range data by sociopolitical level.

**Level**	**Sample size**	**Mean ln[pop. size]**	**s.d.**	**Geomean pop. size**	**95% CL**	**95% CL**
*ω*	*n*	$\ln{\bar{N}}_{\omega }$	*σ*_ln*N*_	${\bar{N}}_{\omega }^{}$	Lower	Upper
1	412	8.55	3.03	5,176	3,863	6,935
2	351	10.15	2.93	25,628	18,866	34,813
3	187	12.08	2.59	177,106	122,230	256,620
4	140	13.48	2.73	713,318	453,540	1,121,893
5	30	14.29	3.81	1,599,611	408,630	6,261,798
**Level**	**Sample size**	**Mean ln[Area]**	**s.d.**	**Geomean area**	**95% CL**	**95% CL**
*ω*	*n*	$\ln{\bar{A}}_{\omega }$	*σ*_ln*A*_	${\bar{A}}_{\omega }^{}$	Lower	Upper
1	412	7.40	2.03	1,643	1,351	1,997
2	351	7.47	2.18	1,754	1,396	2,204
3	187	8.70	2.38	6,004	4,270	8,441
4	140	9.48	2.54	13,094	8,591	19,959
5	30	9.58	3.71	14,432	3,829	54,394

For geographic ranges we define the branching ratio, *R*_*A*_ as:

$$R_{A}=\frac{{\bar{A}}_{\omega +1}^{}}{{\bar{A}}_{\omega }^{}}.$$ (2)

We calculate branching ratios between the five levels. If the branching ratios are constant across all levels then the structure is considered to be statistically self-similar. Rearranging Eqs 1 and 2 we then have exponential functions linking population sizes and geographic ranges to levels of sociopolitical complexity:

$${\bar{N}}_{\omega +k}^{}={\bar{N}}_{\omega }^{}e^{\lambda k}$$ (3)

**Fig 4:**
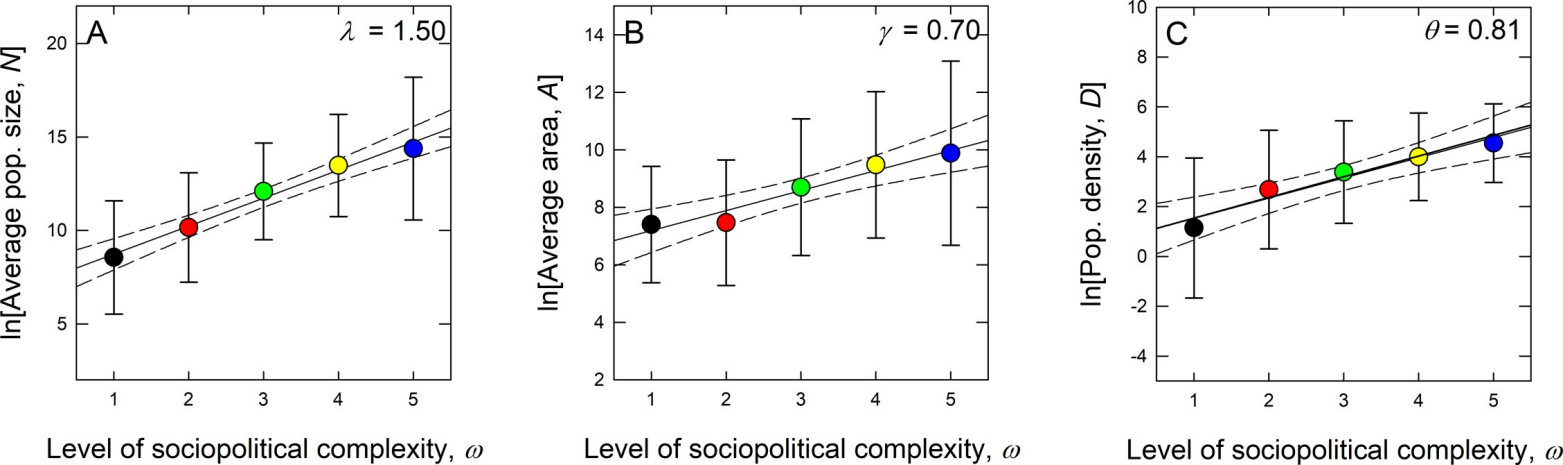
Means (and standard deviations) of ethnolinguistic population sizes (A), geographic ranges (B), and population density (C) by level of sociopolitical complexity. The color coding follows from Fig 1 and is used throughout the paper. The solid black lines are OLS regression fits and the dashed lines are 95% confidence intervals around the slope. Despite the overlap in data among classes in all plots all means are well-fit by the regression models and fall within the 95% confidence intervals. Full results are provided in the S1 File.

And,

$${\bar{A}}_{\omega +k}^{}={\bar{A}}_{\omega }^{}e^{\gamma k}$$ (4)

where *k* = Δ*ω*, *λ* = ln*R*_*N*_ and *γ* = ln*R*_*A*_. Eqs 3 and 4 hypothesize that the average size or geographic range of a population at any one level of complexity is simply the average at another scale multiplied by the appropriate number of branching ratios. To test whether branching ratios are constant across all levels (i.e., statistically self-similar) we plot average population size ${\bar{N}}_{\omega }^{}$, ${\bar{A}}_{\omega }^{}$, and ${\bar{D}}_{\omega }$ as a function of sociopolitical level *ω*. If semi-log plots of $\ln{\bar{N}}_{\omega }^{}$, $\ln{\bar{A}}_{\omega }^{}$, $\ln{\bar{D}}_{\omega }$ and *ω* respectively are well-fit by straight lines then the branching structure is said to be statistically self-similar. These slopes are estimated by ordinary least squares (OLS) regressions in Fig 4 (the statistics of which are given in the S1 File).

**Fig 5:**
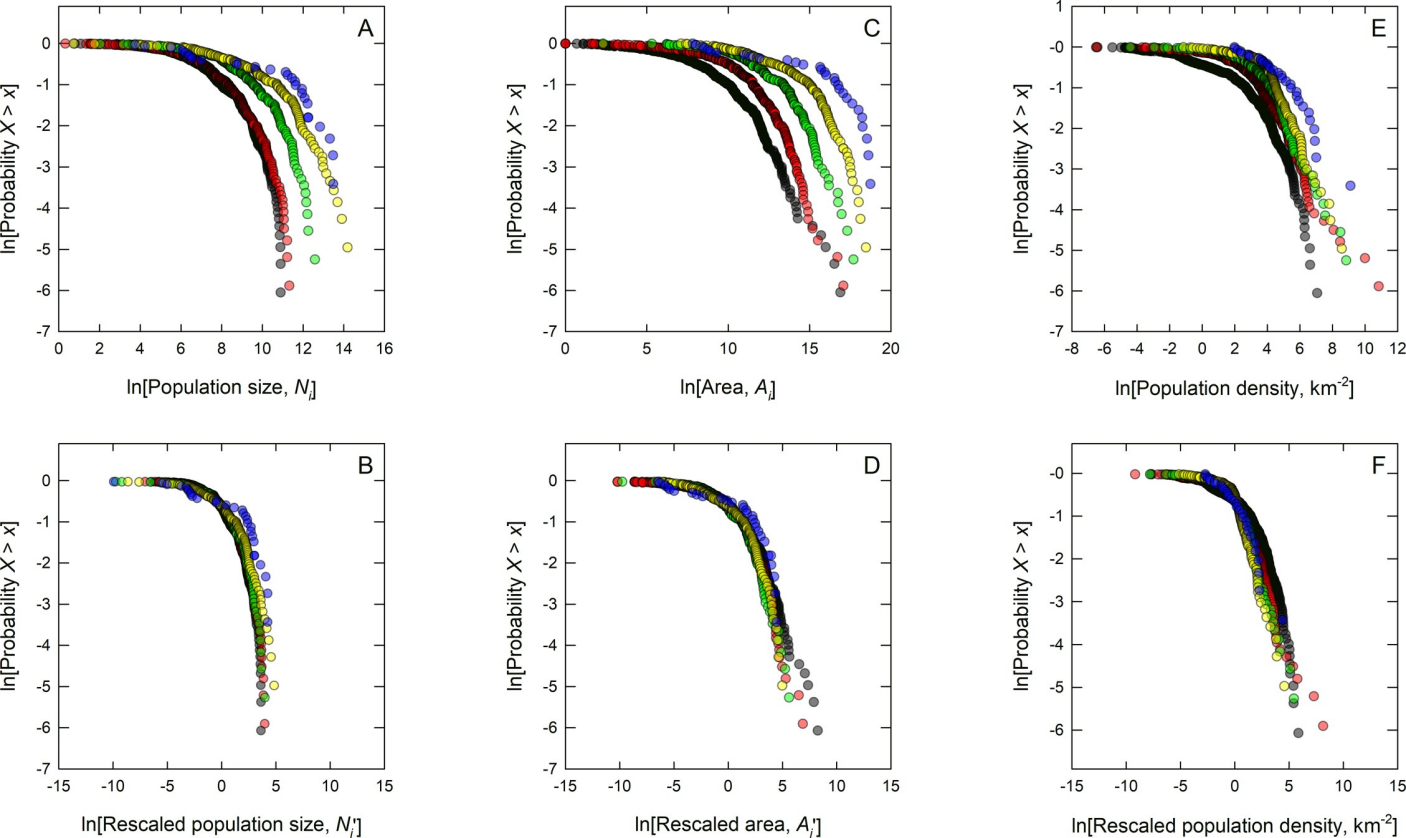
Data collapse of population sizes, geographic ranges, and densities across the five levels of sociopolitical complexity using Generalized Horton Laws: A) Probability distributions of the raw population size data; B) rescaled population size data; C) probability distributions of the raw population geographic range data; and D) rescaled population geographic range data;) E) probability distributions of the raw population density data; and D) rescaled population density data. These distributions are plotted as complementary cumulative distribution functions, *P*_*X*_(*x*) = Pr(*X*>*x*), which plots the probability that some random variable *X* is greater than an observation *x*. Here, we take the probability distributions of *N* and *A* at each level of sociopolitical complexity and rescale them by their respective means. If societies are self-similar across levels of sociopolitical complexity in population size, *N*, geographic range, *A*, and density, *D*, then the probability distributions at each level should collapse onto a single curve when rescaled by their means. Indeed, shows that in all three cases, when rescaled by their means (i.e., non-dimensionalized) all data collapse onto each other indicating that all moments of the distributions are self-similar.

Fig 4A–4C show that the semi-log plots are well-fit by linear functions as all the means are encompassed by the 95% confidence interval around the slope of the OLS models indicating that the means of the distributions are statistically self-similar. Later in the paper we use mixed-effects models to account for variation within each level. In addition, in the S1 File we use quantile regression models to show that this self-similarity is not limited to the means, but is a property of all quantiles of the distributions. Further, Fig 5 shows that this self-similarity is a general property of the entire probability distributions of population metrics across all levels of sociopolitical complexity. This is because when the probability distributions of population size, geographic range, and density are rescaled by their respective means at all levels they collapse onto a single curve, showing that the entire distributions are statistically self-similar [2,52,53].

From Fig 4A the estimated population branching ratio is *R*_*N*_ = exp(*λ*) = 4.47 (3.57−5.44), and from Fig 4B the geographic range branching ratio is *R*_*A*_ = exp(*γ*) = 2.01 (1.66−2.42). Therefore, on average, each level of sociopolitical complexity is associated with an additional level of jurisdictional hierarchy, a four-fold increase in population size, a two-fold increase in spatial extent, and a consequent doubling of population density, as is shown in Fig 4C where *θ* = 0.83 (±0.10), and so exp(*θ*) = 2.25 (1.81−2.80). As both *N* and *A* are functions of scale, *ω*, we can express the change in geographic range as a function of a change in population size by combining Eqs 3 and 4 to find $\bar{A}\propto {\bar{N}}^{\beta }$ where *β* = *γ*/*λ*. While this proportionality is written in terms of averages, the quantile regressions in the S1 File and data collapse of Fig 5 shows that this scaling dynamic is in fact a general property of the entire probability distributions of *A* and *N*. The scaling of population geographic range and population size across levels of sociopolitical complexity is governed by an exponent, *β*, which is predicted to be the ratio of the logarithms of the branching ratios of population geographic ranges and sizes between levels (i.e., *β* = *γ*/*λ* = ln*R*_*A*_/ln*R*_*N*_). As we have empirical estimates of *γ* and *λ* from Fig 4A and 4B, we then have the hypothesis $\bar{A}\propto {\bar{N}}^{0.70/1.50}\propto {\bar{N}}^{0.47}$, which we test and find support for in the S1 File (S2 Fig in S1 File). Thus, our derivation and statistical analysis explicitly links the spatial scaling of population density to the self-similarity of population structure across levels of sociopolitical complexity.

### Mixed model of population density and sociopolitical complexity

The above analysis explored the scaling dynamics of societies across levels of sociopolitical complexity. Now we turn to modeling the scaling of population size and geographic range within each level of sociopolitical complexity, and compare them to see how scaling patterns within each level compare across levels.

To capture the full nature of these dynamics across the entire data set we now build a complete statistical model of sociopolitical complexity across all populations. Each level of sociopolitical complexity, *ω*, is composed of populations that vary in size, *N*, and geographic range, *A*. We express the relationship between the size and geographic range of populations within each level using the standard scaling equation:

$$A_{\omega }=A_{0,\omega }N_{\omega }^{\beta_{\omega }}$$ (5)

where *A*_0,*ω*_ is the area per capita at the *ω*th level (when *N* = 1), and *β*_*ω*_ = *d*ln*A*/*d*ln*N* is the elasticity of a proportional change in geographic range to a change in population size at the *ω*th level. The question of interest here is how the parameters *A*_0,*ω*_ and *β*_*ω*_ in Eq 5 vary across sociopolitical levels as these parameters capture the spatial ecology of populations. In subsistence-level populations, the area per individual *A*_0,*ω*_ is determined primarily by the space required by an individual to meet dietary and resource demands, and the packing of individuals in space, parameterized by *β*_*ω*_ [54,55]. Note that when *β*_*ω*_ = 1, *A* = *A*_0_*N* and so the total area of a population is simply the linear sum of non-overlapping individual areas. However, when *β*≠1, individual areas overlap at a rate *N*^−*β*^. Further, note that by implication from Eq 5, the scaling exponent for each level of complexity, *β*_*ω*_, is the product of the branching structure of social organization within societies.

We model these parameters using a mixed-effects model with random intercepts and slopes. On the log scale, the full model has the general form **Y** = **X***β*+**Z***μ*+*ε* where **X** and **Z** are matrices of data, *β* is a vector of fixed effects, *μ* are the random effects, and $\varepsilon =N\left(0,\sigma_{\varepsilon }^{2}\right)$ is a normal distribution of residuals errors. Specifically, we model the scaling of population density across levels of sociopolitical complexity using a spatial mixed-effects model (spaMM) [56–58], which controls for the spatial autocorrelation of populations, and the evolutionary nesting of ethnolinguistic populations within languages, language families, and continents, for both continuous and categorical variables. Goodness-of-fit is estimated using out-of-sample cross validation (see Methods below). Full details of the model and the complete results are presented in the S1 File.

The full model we fit to the data can be written as follows:

$$\ln A=\ln A_{0}+\beta \times \ln N\times \omega \times \left(1|C\left(F\left(L\right)\right)\right)+M\left(1|longitude+latitude\right)$$ (6)

Where (1|…) denotes a random effect; **M** is a Matérn kernel, which is a covariance matrix of longitudes and latitudes; and *C*, *F*, and *L*, are nested random effects of continent, language family, and language respectively, as described in the Methods section of the main paper. *A* is the geographic range of a population, *N* is the population size, and *ω* is the level of sociopolitical complexity.

**Fig 6:**
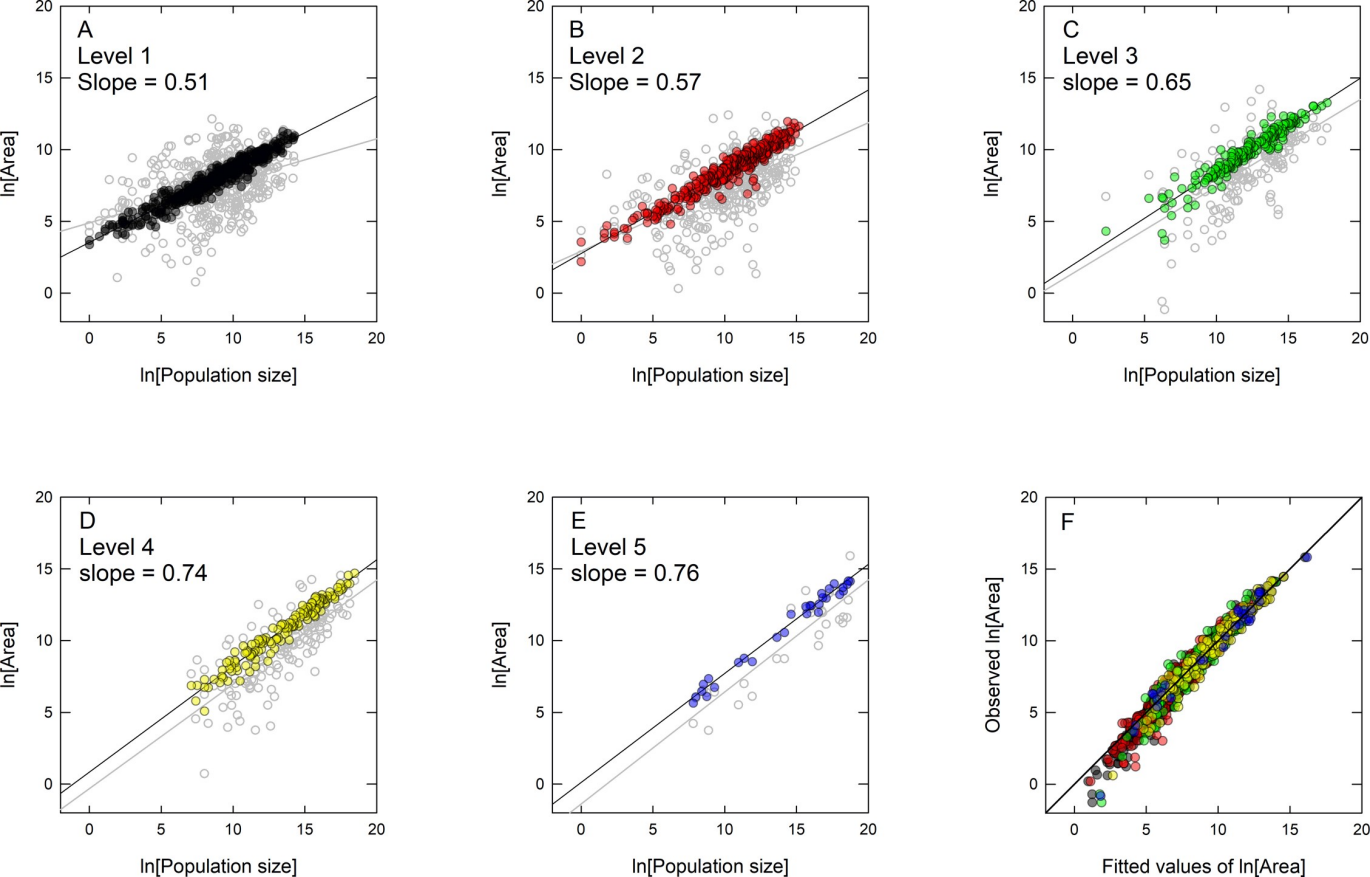
Bivariate plots of the population size-geographic range scaling for each of the five levels of sociopolitical complexity (A-E). The reported slopes are from the mixed-effects model, the results of which are reported in Table 2. The gray data in the background is the original data for each level prior to modeling. This data is shown to illustrate how the mixed-model collapses much of the variation on the *y*-axis at each level, often adjusting the intercepts and slopes, revealing much tighter scaling relationships. F is a plot of the observed vs. expected data from the model, where the *y*-axis is the original data and the *x*-axis is the fitted data. The line is the 1:1 slope along which the data cluster showing the data is well-fit by the model.

**Fig 7:**
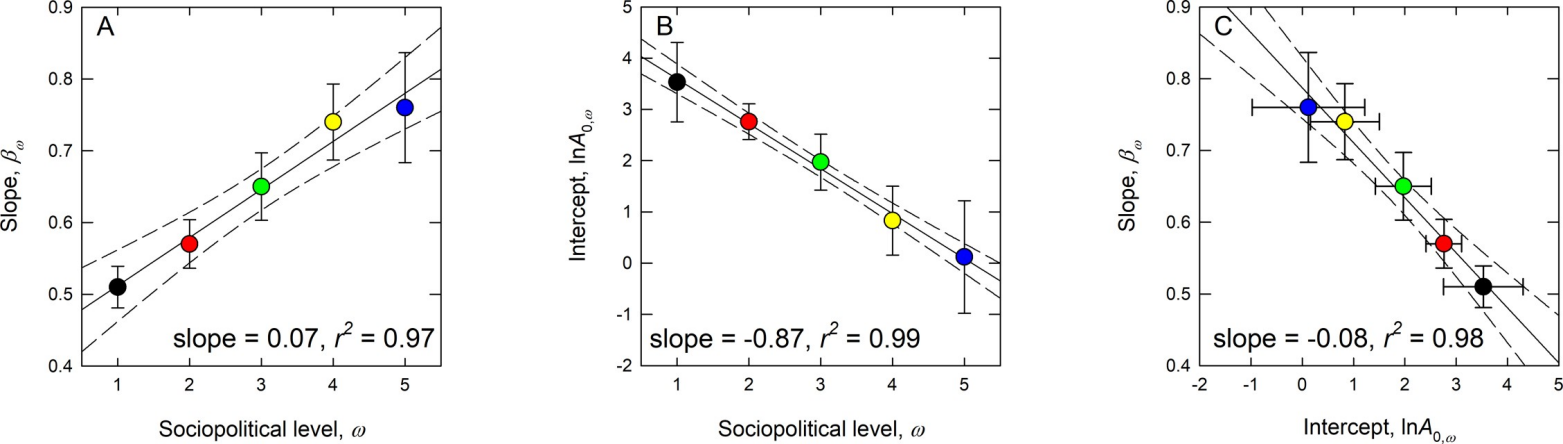
Bivariate plots summarizing the behavior of the slopes and intercepts across the five levels of sociopolitical complexity, as shown in the panels of Fig 6 and Table 2. A) The scaling exponents increase constantly at a rate of 7% with each additional level of sociopolitical complexity. B) The intercepts of the model (the area per individual) decrease by ~42% with each additional level. C) The slopes of the model decrease as the intercepts increase showing how population density is reconfigured at each level of sociopolitical complexity. The color-coding follows from previous figures.

Table 2 reports the results and Fig 6A–6E show the log-log scaling of geographic range, *A*, and population size, *N*, for the five levels of sociopolitical complexity, *ω*. At each additional level of sociopolitical complexity we see an increase in the slope (*β*×ln*N*_*ω*_ in Table 2) and a decrease in the intercept (i.e., factor(Level) *ω* in Table 2 and Fig 7B). Fig 7A shows that the slope *β* increases by 7% with each additional level of complexity, and so populations with increasing levels of sociopolitical complexity have considerably steeper spatial allometries. Fig 7B shows that the intercepts decrease by 42% (exp(−0.87)) with each level of sociopolitical complexity. This means that across the range of sociopolitical complexity there is systematic behavior in the restructuring of population density (Fig 7C); as populations increase in sociopolitical complexity the amount of area per individual decreases rapidly (by 42%) and the degree of spatial packing increases (by 7%).

**Table 2:** Summary of the fixed effects in the spatial mixed-effects model (cross-validated test *R*^2^ = 0.67 (training *R*^2^ = 0.98) with effective d.f. = 401.46; see OSM for full results).

Fixed Effect	Estimate	Cond. SE	t-value*
ln(Intercept)	3.52938	0.7754	4.552
ln*N*	0.51185	0.02927	17.489
factor(Level)2	-0.765	0.34813	-2.197
factor(Level)3	-1.56394	0.54692	-2.86
factor(Level)4	-2.70212	0.67184	-4.022
factor(Level)5	-3.41055	1.09911	-3.103
lnN:factor(Level)2	0.06178	0.03398	1.818
lnN:factor(Level)3	0.1409	0.04691	3.004
lnN:factor(Level)4	0.22464	0.05305	4.234
lnN:factor(Level)5	0.24661	0.07657	3.221

*Note that mixed-effects models use maximum likelihood to estimate parameters and so do not produce *p*-values [59–61]. However, all *t*-values are >2 standard errors or more from the mean, with the exception of lnN:factor(Level2) = 1.812.

## Discussion

In this paper we show two sets of scaling results. First, our results show the self-similarity of population metrics *across* the spectrum of sociopolitical complexity in this global sample of societies. On average, societies at adjacent levels of sociopolitical complexity are characterized by a four-fold difference in population size, a two-fold difference in geographic range, and, therefore, a two-fold difference in population density. Empirically, population densities in the most complex societies can be over 30-times denser than in the least complex societies. However, this pattern is probabilistic, not deterministic. The considerable overlap of population metrics across the range of sociopolitical complexity shown in Fig 4 highlights the statistical nature of this difference, meaning that the differences between levels of sociopolitical complexity cannot be driven solely by demography, but undoubtedly by the interaction of all kinds of endogenous (i.e., demographic, economic, technological, and organizational) and exogenous (environmental, climatic, geographic) mechanisms. While increasingly complex societies tend to be larger and denser the opposite is not true; larger and denser populations are not necessarily more complex.

Self-similarity is further demonstrated by rescaling the entire dataset [62]. Fig 5 shows that the probability distributions of population sizes, geographic ranges, and densities collapse onto single scaling functions when rescaled by their means, thus showing that all these populations are effectively rescaled versions of each other, hence self-similar. The entire distribution of population metrics at any one level of sociopolitical complexity are replicated at all other levels, simply rescaled by a constant, and this rescaling constant is the branching ratio between levels of sociopolitical complexity. So, while societies at different levels of sociopolitical complexity exhibit enormous qualitative diversity in economic, social, and cultural institutions, as well as in their languages, traditions, and norms, the quantitative structure of their organization remains surprisingly invariant.

These results add an additional dimension to the emerging understanding of the importance of self-similarity in human population structure over space and time. It is now well-established that the topological structures *within* human social organizations of all kinds are often self-similar, as local modular clusters are connected to others via multi-tiered interaction networks at constant rates [2,27]. Examples range from hunter-gatherer bands [63] and online gaming networks [41], to networks of traders [43] and self-organized communities of practice [49]. This self-similarity has now been demonstrated in time too. For example, using archaeological data, recent analyses of sociopolitical organization within the world’s first agricultural states finds the same structure [13]. However, here we show that this type of self-similar branching structure holds *across* societies over the wide spectrum of sociopolitical complexity, from hunter-gatherers to state level societies. The branching rates that describe how organizational structure varies across societies with different levels of sociopolitical complexity are remarkably similar to the branching structure observed within societies. Therefore, self-similarity is found both within and across societies.

Second, our results show that as population density doubles across levels of complexity, population size-geographic range scaling evolves *within* levels in interesting ways. Figs 6 and 7 show that with increasing complexity scaling exponents become steeper while intercepts decrease. Thus, the response of population density to size is scale-dependent as population structure is reconfigured in space. This is because increases in sociopolitical complexity are not simply demographic, but are associated with innovations in technology, infrastructure, and lifestyles [24]. A typical individual living in a complex agricultural state will lead a very different lifestyle to an individual living in a hunter-gatherer band and this difference is captured quantitatively by the difference in scaling parameters. By definition, spatial reconfiguration restructures the interactions among individuals within societies of different complexity. Individuals living in denser populations will interact with others more frequently in time and space, and in increasingly complex societies these interactions will be increasingly specialized and structured. Thus, differences in the scaling parameters reflect the fact that populations are not only denser (decreasing the intercepts), but are interacting with each other in space in different ways (steepening the slopes). Recent work in urban scaling shows similar results; as interactions among individuals within settlements become increasingly mediated by more densely built infrastructure, amorphous settlements become increasingly networked, and scaling exponents necessarily steepen [36,64,65]. Our results are also consistent with other studies of human space use, also including the spatial ecology of hunter-gatherers [54,55], agriculturalists [5,66], village level societies [67], both ancient and modern states [68,69], as well as ancient [34,70], medieval [35] and modern cities [36,37,71,72]. In all of these cases, population size increases sublinearly with geographic range indicating that as populations grow in size, they become denser in space.

The causal mechanisms that drive the evolution of sociopolitical complexity over time are contentious. On the one hand, recent research using newly compiled data shows that transitions in social complexity over the Holocene are related to endogenous factors of population growth and information processing mechanisms, as increased demographic scale requires increased organization to maintain stability [58, and see 59]. Interestingly, other research suggests that one of these information thresholds may have been facilitated by the evolution of “moralizing gods”, a collective belief system that helped bind complex multi-ethnic empires at vast geographic scales [25,73,74]. Indeed, new levels of sociopolitical complexity were often accompanied by new roles of pre-existing economic, social, and political institutions [13,15,23,75–82]. On the other hand, other research shows how exogenous factors, such as environmental risk or population pressure influence sociopolitical complexity [83–86]. Human societies are complex systems composed of multiple interacting components, all of which interact with the complex environmental systems on which they rely at multiple scales. As such, the evolution of a trait as complex as sociopolitical organization cannot be driven by a single causative factor [68,87–89]. Ultimately different levels of sociopolitical complexity involve qualitative differences in the form of sociopolitical leadership, infrastructure networks, settlement patterns, technological innovations, productivity, and economic specialization that result from the complex interactions, correlations, and feedbacks that build among systems over time and space. However, the fundamental structure over which these interactions play out is statistically self-similar.

Our results are not inconsistent with either of these positions; we show there is a clear correlation of demographic scale and complexity, but demography cannot be the sole driver given the nature of these data (Fig 4). S5 Fig in the S1 File shows the same data as Fig 7, but highlights both the averages of population size and geographic range (the dashed lines) and the bounding boxes of the range of values within each level of complexity (the colored rectangles). There is a clear tendency for the average size and area of populations to increase (i.e., move up and to the left) with additional levels of sociopolitical complexity, but note the changes in the bounding boxes. The average sizes and areas of populations at any level fall within the bounding boxes at any other level, suggesting that while there may be a statistically significant positive correlation of population size and geographic range with complexity, demographic parameters are not a good discriminator of a society’s level of complexity. Societies of ~6,500–1.5 million occur at all levels of complexity. Therefore, while increasingly complex societies tend to be larger and denser on average than less complex societies, large and dense populations exist at all levels of complexity. The results of our mixed model show that it is not density that is necessarily important, but how that density is configured in space.

Finally, it is important to note that while levels of sociopolitical complexity correlate with different scales of population size, geographic range, and density, the direction of causality remains unclear. First, while there has been a net increase in sociopolitical complexity over the Holocene, this trajectory is not only asymmetric but nonlinear; human societies commonly cycle through periods of growth, stability, and collapse [90,91], often associated with shifts in sociopolitical complexity [24,75,76,92]. And second, political centralization and growing socioeconomic asymmetries impact human societies in complex ways. For example, it could be the case that societies with increasingly formalized sociopolitical infrastructures have a greater capacity for growth and expansion. Or alternatively, it could be the case that societies in riskier environments tend to be more innovative stimulating growth leading to additional levels of sociopolitical hierarchy. Or perhaps there is no clear linear causality [93]; as deeply entangled endogenous and exogenous traits interact to impact the size, density, and organization of societies, complex feedbacks are set in place that, in time, result in a wide diversity of sociopolitical complexity across human societies.

## Methods and data

Ethnolinguistic populations (i.e., spatially-discrete populations of language speakers) are among the largest scales of human social organization. Our primary unit of analysis is the ethnolinguistic geographic range, *A*, which is a spatially and linguistically discrete region of the planet’s surface measured in units of km^2^ and inhabited by *N* individuals. Sizes and geographic ranges vary widely, from a handful of speakers covering an area of a few square kilometers, to many millions of speakers covering hundreds of thousands of square kilometers. Multiple ethnolinguistic populations may share a common language, *L*. Ethnolinguistic geographic range polygon shapefiles (*N* = 7,627) and population sizes were downloaded from the *Ethnologue* [94] and we matched these polygons with the *Ethnographic Atlas* to find their traditional level of sociopolitical complexity. For each ethnolinguistic polygon we first searched for direct matches with language names in the *Ethnographic Atlas* [21]. For every ethnolinguistic polygon with no direct match with the *Ethnographic Atlas* we then conducted an online search through the ethnographic literature for alternative names, alternate spellings, or tribal affiliations. We were able to match 1,284 ethnolinguistic polygons from the *Ethnologue* with the societies listed in the *Ethnographic Atlas*. We made a total of 964 total matches between individual language names across data sets, but as these languages are sometimes spoken in multiple ethnolinguistic populations, the total number of polygons increased to 1,121. To control for this clustering, we used language name, *L*, as a random effect in our models. Using the *Ethnographic Atlas*, for each ethnolinguistic geographic range we recorded language name, *L*, language family, *F*, the continent on which it occurs, *C*, population size, *N*, geographic range, *A*, and the level of sociopolitical complexity, *ω*. Because languages are often spoken by more than one ethnolinguistic population, the level of sociopolitical hierarchy often varies within language families.

To analyze these data, we used a combination of Horton-Strahler branching, generalized Horton Laws, and scaling approaches, as outlined above and in more detail in the S1 File attached to this paper. Horton-Strahler analysis is a commonly-used technique to characterize the hierarchical branching structure of complex networks across the sciences [53]. Each sociopolitical level is assigned a hierarchical order, *ω*, where *ω*∈(1,5), which in Horton analysis is termed the Horton order. Each ethnolinguistic population is then assigned to the sociopolitical level, *ω*, as given by the *Ethnographic Atlas* [21]. In the *Ethnographic Atlas* the level of sociopolitical complexity comes from variable 33 “Jurisdictional Hierarchy Beyond Local Community”, which Murdoch defines as the level of sociopolitical complexity, ranging from 1–5, where 1 = no political authority beyond community; 2 = simple chiefdoms; 3 = complex chiefdoms; 4 = early states; and 5 = large states. The integers refer to the levels of jurisdictional hierarchy. Population size, area, and density are then analyzed throughout the analysis using these levels as identifiers of the level of sociopolitical complexity.

Scaling models were constructed using spatial mixed-effect models, or spaMMs [56–58] and run in R [95]. Here, the dependent variable was geographic range, *A*, and the independent variable was an ethnolinguistic population of size, *N*. The data were normalized by taking the natural logarithms (see S1 File for details). There is no potential of multicollinearity in the data as there is only one independent variable. Each ethnolinguistic population has a level of sociopolitical complexity, *ω*, speaks a language, *L*, (which may or may not be common to other ethnolinguistic populations), nested within a language family, *F*, that is nested within a continent, *C*, which are all potentially correlated in space. The spatial mixed effects models model scaling relationships while controlling for the spatial-autocorrelation of both continuous and discrete variables, which themselves are hierarchically nested. To estimate goodness-of-fit statistics we used out-of-sample cross validation. Here, the data is randomly divided into two sections; a training set of 70% of the data, and a test set of 30% of the data. The statistical models are built using the training set and are then evaluated on their ability to predict the out-of-sample test data. Data and results are available in the online S1 File with the exception of the shapefiles, which unfortunately are behind a paywall: (https://www.ethnologue.com/product/ethnologue-global-dataset-0).

We provide more details, results and analyses in the S1 File associated with this paper.

## Supporting information

S1 File (DOCX)

S1 Data (XLSX)

S1 Rcode (R)
