# Supplementary material for: Scaling human sociopolitical complexity
Source: PLoS One. 2020 Jul 2;15(7):e0234615. doi: 10.1371/journal.pone.0234615 (PMC7332085; doi:10.1371/journal.pone.0234615)
Supplement: S1 File — (DOCX) [file pone.0234615.s001.docx]

Online supplementary materials:

Scaling human sociopolitical complexity

Marcus J. Hamilton^1,2^,*

Robert S. Walker^3^

Briggs Buchanan^4^

David S. Sandeford^5^

^1^ Department of Anthropology, University of Texas at San Antonio, San Antonio, TX

^2^ Santa Fe Institute, Santa Fe, NM

^3^ Department of Anthropology, University of Missouri, Columbia, MO

^4^ Department of Anthropology, University of Tulsa, Tulsa, OK

^5^ School of Human Evolution and Social Change, Arizona State University, Tempe, AZ

* Corresponding author

In the following document we provide further information, details, and discussion of the statistical tests and modeling performed in the paper, as well as conducting additional tests to clarify and generalize our results.

**Data distributions.** We start by considering the distributions of the raw data, and normalization through log-transforming. In Figure S1, we plot the fifteen distributions of the population sizes, areas, and densities at the five levels of complexity on the linear scale.





**Figure S1.** Frequency distributions of the three main categories of data used in the paper across the five levels of sociopolitical complexity plotted on the linear scale.

All 15 distributions are highly non-normal and heavily skewed to the left, as shown in Figure S1. We log-transformed the data to normalize the distributions, as shown in Figure 3 of the paper. Table S1 gives the *P*-values of this normalization (using Shapiro-Wilks normality tests), indicating that log-transforming successfully normalizes the data (14 out of 15 distributions have $P>\alpha=0.01$), so we are confident in using data on the log-scale in the rest of the analyses. Note that formal normality tests are quite conservative and so are prone to false positive (type I errors). As such, significance values should be viewed with caution.

Table S1. Results of Shapiro-Wilks normality tests for the frequency distributions in Figure 3 (*a* = 0.01). The only distribution significantly different from lognormal is level 2 population density.

| **Level** | **lnPopulation size** | **lnPopulation area** | **lnPopulation density** |
| --- | --- | --- | --- |
| 1 | P = 0.09 | P = 0.03 | P = 0.73 |
| 2 | P = 0.12 | P = 0.09 | P < 0.01 |
| 3 | P = 0.19 | P = 0.10 | P = 0.06 |
| 4 | P = 0.22 | P = 0.01 | P = 0.01 |
| 5 | P = 0.01 | P = 0.04 | P = 0.01 |

**
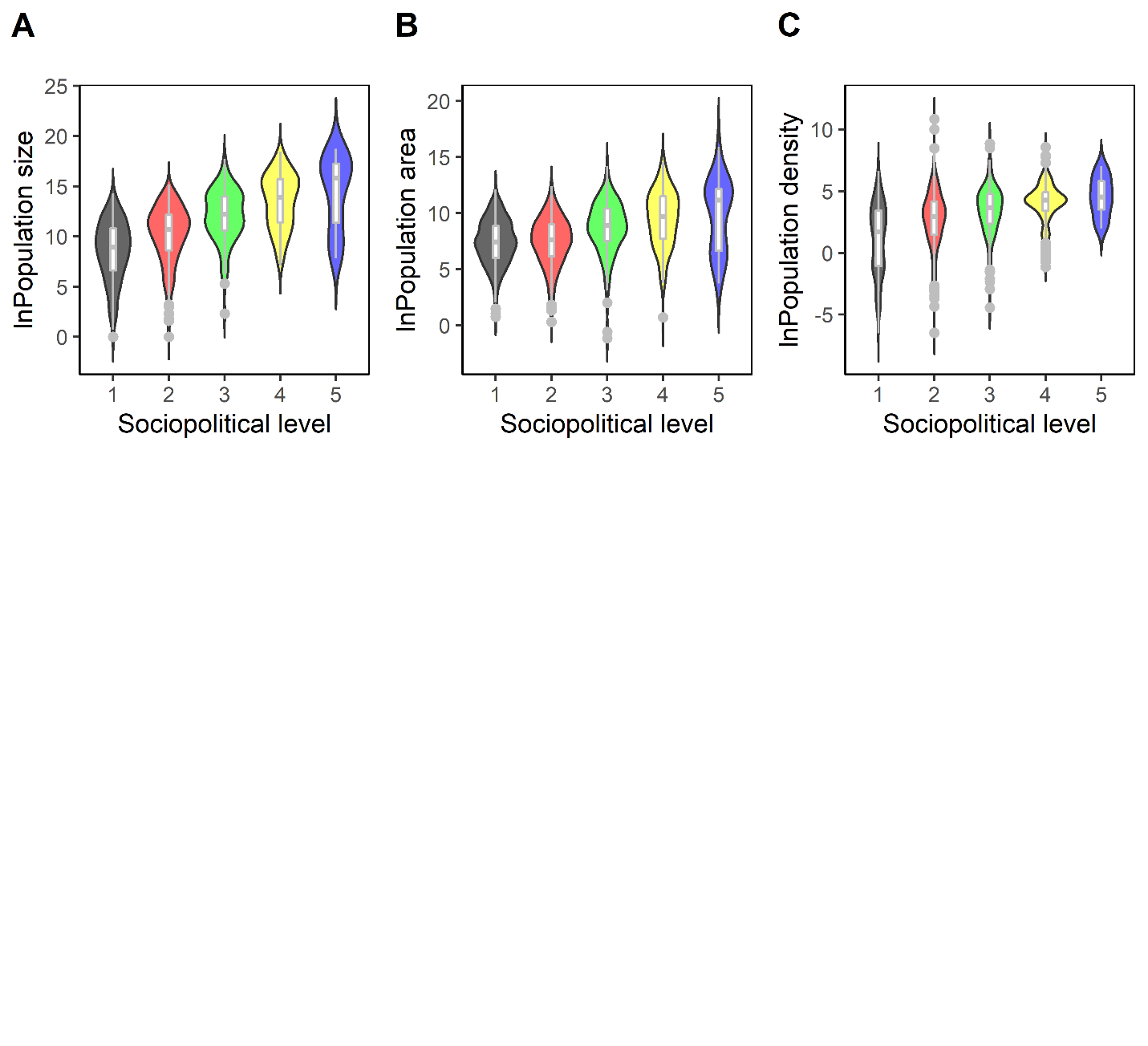
**

**Figure S2.** Violin plots of the raw data by sociopolitical level.

Figure S2 is a violin plot of the raw data illustrating the spread and distribution on the data.

**Regression analyses of the Horton-Strahler branching ratios.** In the first statistical tests of the paper we use OLS regression to test whether the average size, areas, and densities of populations across the five levels of sociopolitical complexity are well-fit by linear functions. This is tested in two ways. The first is simply whether all data points fall within the 95% confidence limits of a fitted OLS function. In Figure 4 of the main paper shows that all data points fall within these limits. Below we give the regression statistics for each hypothesis test and show that all regressions are significant, as are the tests of the normality of the residuals and constant variance. This is the first statistical test that shows the branching rates between the levels of sociopolitical complexity are statistically self-similar. The following results are presented in the sequence they appear in the paper; Figures 4A, B, and C:

**Regression of population means (Figure 4A):**

R Rsqr Adj Rsqr Standard Error of Estimate

0.9924 0.9848 0.9797 0.3403

Coefficient Std. Error t P

y0 7.2342 0.3569 20.2682 0.0003

a 1.4983 0.1076 13.9223 0.0008

Analysis of Variance:

DF SS MS

Regression 2 710.3036 355.1518

Residual 3 0.3474 0.1158

Total 5 710.6511 142.1302

Corrected for the mean of the observations:

DF SS MS F P

Regression 1 22.4484 22.4484 193.8316 **0.0008**

Residual 3 0.3474 0.1158

Total 4 22.7959 5.6990

Statistical Tests:

Normality Test (Shapiro-Wilk) Passed (P = 0.6319)

W Statistic= 0.9352 Significance Level = 0.0500

Constant Variance Test Passed (P = 0.0500)

**Regression of population areas (Figure 4B):**

R Rsqr Adj Rsqr Standard Error of Estimate

0.9722 0.9451 0.9268 0.3066

Coefficient Std. Error t P

y0 6.4969 0.3216 20.2047 0.0003

a 0.6968 0.0970 7.1873 0.0056

Analysis of Variance:

DF SS MS

Regression 2 373.5691 186.7845

Residual 3 0.2820 0.0940

Total 5 373.8511 74.7702

Corrected for the mean of the observations:

DF SS MS F P

Regression 1 4.8556 4.8556 51.6569 **0.0056**

Residual 3 0.2820 0.0940

Total 4 5.1376 1.2844

Statistical Tests:

Normality Test (Shapiro-Wilk) Passed (P = 0.2036)

W Statistic= 0.8528 Significance Level = 0.0500

Constant Variance Test Passed (P = 0.0500)

**Regression of population density (Figure 4C):**

R Rsqr Adj Rsqr Standard Error of Estimate

0.9732 0.9472 0.9296 0.3501

Coefficient Std. Error t P

y0 0.7161 0.3671 1.9506 0.1462

a 0.8121 0.1107 7.3359 0.0052

Analysis of Variance:

DF SS MS

Regression 2 56.2797 28.1399

Residual 3 0.3676 0.1225

Total 5 56.6473 11.3295

Corrected for the mean of the observations:

DF SS MS F P

Regression 1 6.5944 6.5944 53.8156 **0.0052**

Residual 3 0.3676 0.1225

Total 4 6.9620 1.7405

Statistical Tests:

Normality Test (Shapiro-Wilk) Passed (P = 0.7269)

W Statistic= 0.9486 Significance Level = 0.0500

Constant Variance Test Passed (P = 0.0500)

**Quantile regressions of Horton-Strahler branching ratios.** Now we have established the self-similarity of the geometric means of population size, area, and density across levels of sociopolitical complexity, we now show this is a more general property across the range of quantiles of all the distributions. That is to say, not only is self-similarity apparent near the mode or 50^th^ percentile of the distributions, but also at all others. To show this we use quantile regression [1]. We run regressions through all 10% quantiles, from 10%-90% across all three distributions. We present the summarized R output for population size, area, and then density below, and we summarize the results visually in Figure S2. All quantiles across all distributions are highly significant ($P\ll\alpha=0.05$). In Figure S2A and D we see the slopes of the regressions are relatively constant across quantiles. The red lines in Figures S2D-F are the OLS estimates reported in the main text (Figure 4), and the dark grey lines are the averages of the quantile slopes. In all three cases they are very similar. In Figure S2B and E we see that regressions slopes are shallowest at the lowest quantiles of geographic ranges, and steepest at the highest. As a result, in Figures S2C and F we see that the slopes in population densities are highest at the lowest quantiles, and shallowest at the highest quantiles. Therefore, branching ratios between population densities at differing levels of sociopolitical complexity are highest in the lowest densities at each level and lowest at the highest densities per level. However, the average of the quantile estimates are similar to the OLS estimates found in the analysis of geometric means.





**Figure S3.** Bivariate plots of quantile regressions of population structure across levels of sociopolitical complexity.

**Quantile regression output for population sizes**

Coefficients:

tau Value S.E. t-value Pr(>|t|)

(Intercept) 0.1 2.67728 0.44347 6.03718 0.00000

Level 0.1 1.70475 0.1869 9.12105 0.00000

(Intercept) 0.2 4.54629 0.37732 12.04887 0.00000

Level 0.2 1.65849 0.15815 10.48663 0.00000

(Intercept) 0.3 5.84195 0.28892 20.21982 0.00000

Level 0.3 1.57262 0.11957 13.15193 0.00000

(Intercept) 0.4 6.69971 0.25913 25.85481 0.00000

Level 0.4 1.59184 0.11316 14.06757 0.00000

(Intercept) 0.5 7.28245 0.24252 30.02834 0.00000

Level 0.5 1.65709 0.10986 15.08388 0.00000

(Intercept) 0.6 7.9371 0.2259 35.13494 0.00000

Level 0.6 1.72431 0.09116 18.91495 0.00000

(Intercept) 0.7 8.65824 0.19853 43.61136 0.00000

Level 0.7 1.68075 0.07187 23.3856 0.00000

(Intercept) 0.8 9.52077 0.23262 40.92758 0.00000

Level 0.8 1.60649 0.07651 20.99705 0.00000

(Intercept) 0.9 10.5634 0.17842 59.20612 0.00000

Level 0.9 1.5373 0.07798 19.71379 0.00000

**Quantile regression output for geographic ranges**

Coefficients:

tau Value S.E. t-value Pr(>|t|)

(Intercept) 0.1 4.29717 0.27104 15.85437 0.00000

Level 0.1 0.3621 0.12844 2.8191 0.00490

(Intercept) 0.2 5.14543 0.22296 23.07815 0.00000

Level 0.2 0.46447 0.10962 4.23707 0.00002

(Intercept) 0.3 5.66034 0.19975 28.33698 0.00000

Level 0.3 0.59511 0.09592 6.2043 0.00000

(Intercept) 0.4 6.08847 0.17889 34.03568 0.00000

Level 0.4 0.68666 0.0876 7.83831 0.00000

(Intercept) 0.5 6.4772 0.16729 38.71815 0.00000

Level 0.5 0.7877 0.08054 9.77969 0.00000

(Intercept) 0.6 6.8655 0.15822 43.39076 0.00000

Level 0.6 0.83726 0.07279 11.50277 0.00000

(Intercept) 0.7 7.38968 0.19954 37.03439 0.00000

Level 0.7 0.8519 0.08118 10.49365 0.00000

(Intercept) 0.8 8.17663 0.17022 48.03702 0.00000

Level 0.8 0.81653 0.06622 12.33006 0.00000

(Intercept) 0.9 8.86076 0.20344 43.55495 0.00000

Level 0.9 0.83965 0.08495 9.88453 0

**Quantile regression output for population densities**

Coefficients:

tau Value S.E. t-value Pr(>|t|)

(Intercept) 0.1 -3.6911 0.53233 -6.93394 0.00000

Level 0.1 1.3037 0.18973 6.87151 0.00000

(Intercept) 0.2 -2.4285 0.24134 -10.06262 0.00000

Level 0.2 1.3591 0.10415 13.0492 0.00000

(Intercept) 0.3 -1.2453 0.25373 -4.90829 0.00000

Level 0.3 1.2522 0.07699 16.26482 0.00000

(Intercept) 0.4 0.0654 0.20353 0.32143 0.74795

Level 0.4 0.9935 0.0473 21.00752 0.00000

(Intercept) 0.5 1.1430 0.18798 6.08077 0.00000

Level 0.5 0.7930 0.05007 15.8387 0.00000

(Intercept) 0.6 2.0233 0.16052 12.60455 0.00000

Level 0.6 0.6383 0.05169 12.34827 0.00000

(Intercept) 0.7 2.5127 0.16095 15.61226 0.00000

Level 0.7 0.6084 0.05642 10.78331 0.00000

(Intercept) 0.8 3.2750 0.16372 20.00473 0.00000

Level 0.8 0.5297 0.06108 8.67233 0.00000

(Intercept) 0.9 4.1107 0.20308 20.24133 0.00000

Level 0.9 0.4993 0.07274 6.86412 0.00000

**Generalized Horton analysis and data collapse.** After showing the self-similarity of sociopolitical complexity through the Horton analysis of branching ratios in Figure 5 and the quantile regressions above, we move onto generalized Horton analysis (otherwise known as dynamic scaling). This is a technique of data collapse, and is considered the strongest test of self-similarity [2]. We now know both the geometric means (the first moments of the distributions) are self-similar, as are all quantiles of the distribution. That is to say we could collapse these means and quantiles onto each other by rescaling by a single variable, in this case the slope of the exponential function in equations 3 and 4, which is the branching ratio. Now we ask a much more powerful question; do *all* moments of all distributions collapse onto each other in the same way when rescaled by the same process? If so, the entire distributions are self-similar, not just the means and quantiles.

We begin in Figures 5A, C, and E, by plotting the data as cumulative distribution functions, $P\left(x\right)=P\left(X\right)>x$. These distributions plot all data points without any binning showing all the characteristics of data, and so are a preferred method of data visualization [3] . These plots provide the probability of observing a random variable greater than *x* as you move along the *x*-axis. This probability is 1 when starting at the far left (the minimum size) as it is certain you will observe a value greater than the minimum, and then decays to the right at some rate, and this rate characterizes the probability distribution. Figures 5A, C, and E show that population sizes, areas and densities at different levels of sociopolitical complexity are distributed quite evenly along the x-axis. This indicates that the probability of observing a larger population size, area, or density, at any level is similar, and only seems to differ by some scalar separating the distributions along the x-axis. If indeed all these data points in these distributions are fundamentally the same (i.e., self-similar), then they are separated along the x-axis by a constant value, which we have already hypothesized to be the branching ratio recovered in Figure 4. Therefore, the way to test this is to rescale (or non-dimensionalize) the data by standardizing each value by the average, and by doing so all the distributions should collapse onto each other.

The procedure is as follows: Let $X_{i\omega}$ be the population size (or geographic range, or density) of the *i*th population at the level $\omega$. Each variable is then rescaled by removing the mean of the variable within a level, and so we have $X_{i\omega}'=X_{i\omega}/\overline{X}_{\omega}$ and so the rescaled population size is the original size divided by the average size of a population at a particular level of complexity. This is the done for all data points. All rescaled data are then replotted as cumulative distributions functions, as before. Figures 5B, D, and F show the results of data collapses of population size, area and density. All data collapse onto single scaling functions when rescaled by their means providing strong evidence that these probability distributions are indeed self-similar.

**Scaling of average population size and geographic range.** From the derivation of equation 5 we then show that the average geographic range of a population per level should scale with the average population size per level at a rate determined by the ratio of the logarithmic branching ratios, and so we predict $\beta=0.47$, which we find support for here with regression, and show in Figure S3.

**Regression of average population areas and sizes (Figure S3):**

R Rsqr Adj Rsqr Standard Error of Estimate

0.9756 0.9518 0.9358 0.2836

Coefficient Std. Error t P

y0 3.2141 0.7079 4.5401 0.0200

a 0.4572 0.0594 7.7004 0.0046

Analysis of Variance:

DF SS MS

Regression 2 372.6339 186.3170

Residual 3 0.2413 0.0804

Total 5 372.8753 74.5751

Corrected for the mean of the observations:

DF SS MS F P

Regression 1 4.7700 4.7700 59.2968 **0.0046**

Residual 3 0.2413 0.0804

Total 4 5.0113 1.2528

Statistical Tests:

Normality Test (Shapiro-Wilk) Passed (P = 0.6848)

W Statistic= 0.9427 Significance Level = 0.0500

Constant Variance Test Passed (P = 0.0500)

**

**

**Figure S3.** Bivariate plot of the scaling of average population size and average area over the five levels of sociopolitical complexity.

**Full results of the Spatial mixed-effect model (spaMM).** The complete scaling behavior of the data are captured by the full spatial mixed-effects model reported in the main text. Here we provide the full R output from fitting the spatial mixed-effects model to our data following equation 7 in the main text, including estimates of the random effects. These results can be replicated with the available data and code:

formula: lnA ~ lnN * factor(Level) * (1 | Continent/Family/Language) +

Matern(1 | Longitude + Latitude)

REML: Estimation of corrPars, lambda and phi by REML.

Estimation of fixed effects by ML.

Estimation of lambda and phi by 'outer' REML, maximizing p_bv.

Family: gaussian ( link = identity )

------------ Fixed effects (beta) ------------

Estimate Cond. SE t-value

(Intercept) 3.52938 0.77540 4.552

lnN 0.51185 0.02927 17.489

factor(Level)2 -0.76500 0.34813 -2.197

factor(Level)3 -1.56394 0.54692 -2.860

factor(Level)4 -2.70212 0.67184 -4.022

factor(Level)5 -3.41055 1.09911 -3.103

lnN:factor(Level)2 0.06178 0.03398 1.818

lnN:factor(Level)3 0.14090 0.04691 3.004

lnN:factor(Level)4 0.22464 0.05305 4.234

lnN:factor(Level)5 0.24661 0.07657 3.221

--------------- Random effects ---------------

Family: gaussian ( link = identity )

--- Correlation parameters:

4.nu 4.rho

0.19811434 0.01513576

--- Variance parameters ('lambda'):

lambda = var(u) for u ~ Gaussian;

Language:. : 0.03147

Family:Co. : 0.2145

Continent : 0.6509

Longitude. : 3.807

# of obs: 1120; # of groups: Language:., 802; Family:Co., 100; Continent, 5; Longitude., 1119

------------- Residual variance -------------

phi estimate was 0.586427

------------- Likelihood values -------------

logLik

p_v(h) (marginal L): -1950.410

p_beta,v(h) (ReL): -1965.082

> AIC(scalingmodel)

marginal AIC: 3934.8204

conditional AIC: 3301.2099

dispersion AIC: 3944.1640

effective df: **401.4614**

**Cross-validation procedures:** R output from the out-of-sample cross-validation that we used to estimate the model’s goodness of fit ($R^{2}$). Again, the code is provided. For the cross-validation procedure we ran the same model as above but used a 70/30 split for the training and testing data respectively. The sample size for the training data was 796 and for the test data 332. Mixed models do not produce $R^{2}$ statistics and so cross-validation is one method of evaluating the ability of a model to predict patterns in unseen data. The model is run on the 70% training data, and statistics are collected. The model is then exposed to the unseen 30% test data (hence out-of-sample), and statistics are collected. Therefore, this cross-validation technique provides to $R^{2}$ estimates: one for the training data (i.e., how well the model performed on the training data) and the second for the testing data (i.e., how well the model performed on predicting the test data). Both $R^{2}$ estimates are informative when evaluating model performance.

The R following output can be reproduced using the available data and code:

> # Cross-validation procedure

> postResample(predict(scalingmodel, testN, allow.new.levels = TRUE), testN$lnA)

RMSE Rsquared MAE

1.3571339 **0.6660129** 1.0147426

> postResample(predict(scalingmodel, trainN, allow.new.levels = TRUE), trainN$lnA)

RMSE Rsquared MAE

0.3620116 **0.9810135** 0.2771005

To further visualize the results of the model, below in Figure S4 we provide bivariate plots of the scaling relationships including each of the five levels of sociopolitical complexity (decomposed by each level in Figures 6A-E of the main text), both in terms of the raw data and the modeled data. The figure further illustrates how variation along the Y-axis in the raw data is collapsed by the mixed-effects model revealing much tighter scaling relations than observed in the raw data.

**

**

**Figure S4.** Log-log bivariate plots of the population size and geographic range (area) of the 1,128 societies represented in the dataset over the fivel levels of sociopolitical complexity. A shows the original raw data; B) shows the modeled data; and C) shows the observed vs expected fits of area.

Finally, in Figure S5 we emphasize means and ranges of population sizes and geographic ranges across the levels of sociopolitical complexity. Average population sizes and geographic ranges increase with sociopolitical complexity, but the bounding boxes covering the ranges on data show considerable overlap at all levels. This overlap demonstrates that while more complex societies tend to be larger in size and area than less complex societies, large and dense populations occur at all levels of complexity.


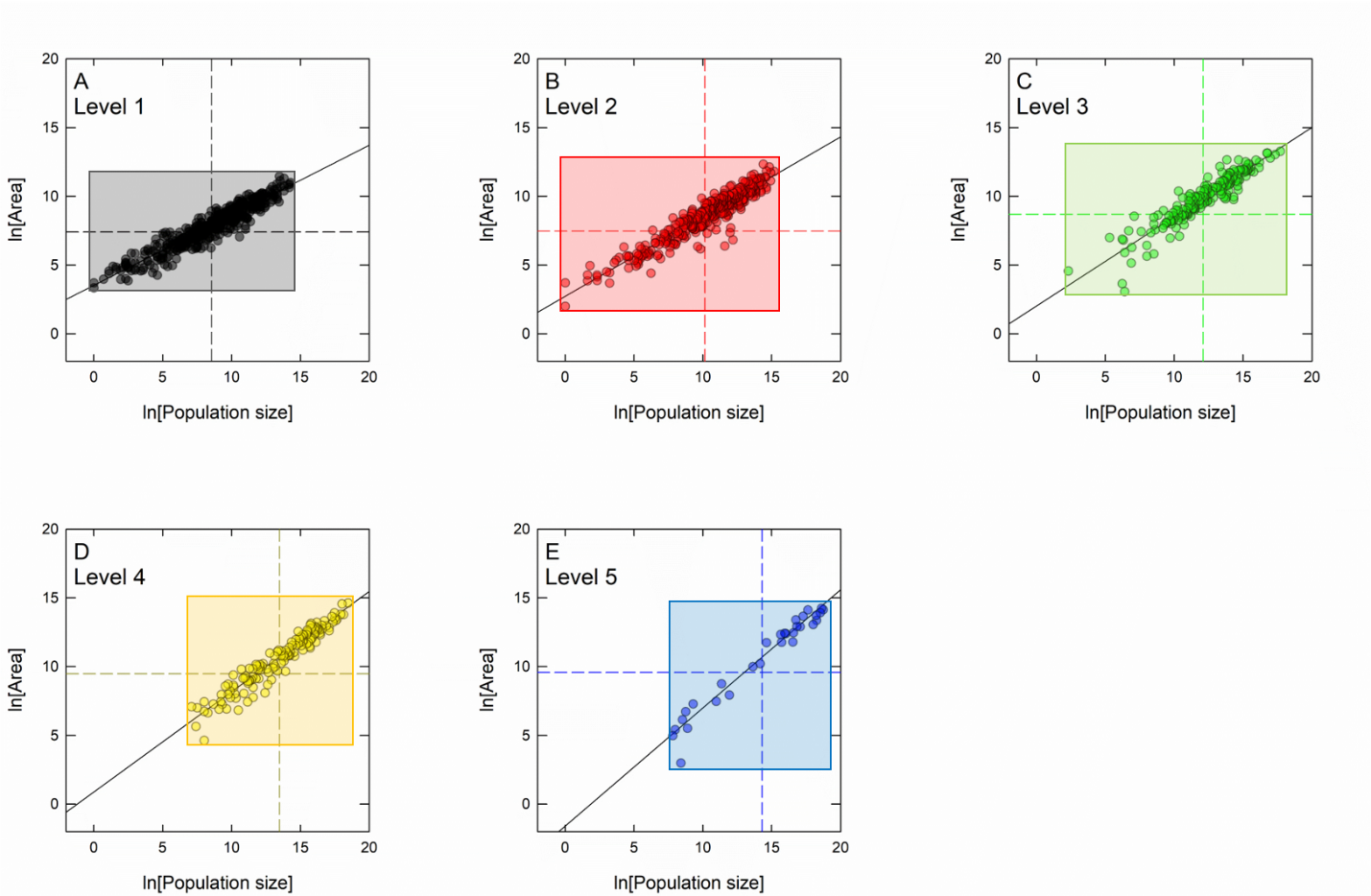


**Figure S5.** Log-log plots of bivariate scaling relationships between population size and geographic range by level of sociopolitical complexity highlighting data averages (dashed lines) and data ranges (bounding boxes).
